# Antiviral potential of *Corchorus olitorius* fixed oil loaded into poly(D,L-lactide-co-glycolide)/poly(ε-caprolactone) (PLGA/PCL) nanoparticles against HSV-1 virus, supported by network analysis

**DOI:** 10.1038/s41598-025-15718-x

**Published:** 2025-11-13

**Authors:** Khayrya A. Youssif, Mohammed H. Elkomy, Sammar Fathy Elhabal, Fatma Mohamed Abd El-Mordy, Mohamed Hisham, Rehab H. Abd El-Aleam, Saeed Abdul kareem Saeed Al-Zuhairy, Mohamed A. el-Nabarawi, Adam A. Al-Shoubki, Arwa Ramadan El-Manakhly, Nesreen A. Safwat, Gerhard Bringmann, Usama Ramadan Abdelmohsen, Nourhan Hisham Shady

**Affiliations:** 1 Department of Pharmacognosy, Faculty of Pharmacy, El Salehya El Gadida University, El Sharquia, El Salehya El Gadida, Egypt; 2 Department of Pharmaceutics, College of Pharmacy, Jouf University, 72341 Sakaka, Saudi Arabia; 3 Department of Pharmaceutics and Industrial Pharmacy, Faculty of Pharmacy, Modern University for Technology and Information (MTI), Mokattam, Cairo, 11571 Egypt; 4 Department of Pharmacognosy and Medicinal Plants, Faculty of Pharmacy (Girls), Al-Azhar University, Cairo, 11754 Egypt; 5 Department of Pharmaceutical Chemistry, Faculty of Pharmacy, Deraya University, New Minia, 61111 Egypt; 6 Department of Pharmaceutical Chemistry, Faculty of Pharmacy, Faculty of Pharmacy, Modern University for Technology and Information (MTI), Mokattam, Cairo, 11571 Egypt; 7 Department of Pharmacy, Kut University College, Kut, Wasit 52001 Iraq; 8 Department of Pharmaceutics and Industrial Pharmacy, Faculty of Pharmacy, Cairo University, Cairo, Egypt; 9 Department of Pharmaceutics and Industrial Pharmacy, Faculty of Pharmacy, University of Derna, Derna, Libya; 10 Department of Pharmaceutics and Industrial Pharmacy, Faculty of Pharmacy, Cairo University, Cairo, 11562 Egypt; 11 Department of Microbiology and Immunology, Faculty of Pharmacy, Modern University for Technology and Information (MTI), Mokattam, Cairo, 11571 Egypt; 12 Institute of Organic Chemistry, University of Würzburg, Am Hubland, 97074 Würzburg, Germany; 13 Department of Pharmacognosy, Faculty of Pharmacy, Deraya University, New Minia City, 61111 Egypt; 14 Deraya Center for Research and Sustainability, Deraya University, Universities Zone, New Minia City, 61111 Egypt

**Keywords:** *Corchorus olitorius*, Fixed oil, Polylactide-co-glycolide (PLGA), Poly(ε-caprolactone) (PLC), Anti-viral, Herpes simplex type 1 (HSV-1), Network pharmacology analysis, Molecular docking study, Computational biology and bioinformatics, Drug discovery, Plant sciences, Chemistry, Nanobiotechnology

## Abstract

Although *Corchorus olitorius* has been traditionally used in folk medicine for various purposes for a long time and has components that may have antiviral effects, there is not much scientific data to support any particular antiviral effects of the plant. More research is required to investigate its antiviral effectiveness against certain viral infections and to comprehend the underlying mechanisms.The goals of this research were to create biodegradable and partially soluble polymeric nanoparticles (polylactide-co-glycolide- poly(ε-caprolactone) nanoparticles) (PLGA-PCL NPs) loaded with *C. olitorius* (referred to as C.O./PLGA-PCL-NPs) and to examine their potential impact against the Herpes simplex type 1 (HSV-1) virus. Gas chromatography coupled with mass spectrometry (GC-MS) was used to chemically characterize the fixed oil of the shrub *Corchorus olitorius* (*C. olitorius*, family Malvaceae). Additionally, we used all the components identified in an in silico molecular docking investigation, screening them against HSV-1 DNA polymerase (PDB ID: 2GV9) and thymidine kinase from HSV-1 complexed with 5-iododeoxyuridine (HSV-1 TK; PDB ID: 1KI7). The possibility of using *Corchorus olitorius* oil (C.O.) to cure Herpes simplex virus type 1 (HSV-1) is examined in this study. C.O., a plant oil rich in fatty acids and sterols, was encapsulated in nanoparticles (NPs) made from a blend of polylactide-co-glycolide (PLGA) and poly(ε-caprolactone) (PCL) polymers. We optimized the formulation to create NPs with minimal clumping, a small size, and a stable electrical charge. The optimized NPs displayed a spherical morphology with minimal agglomeration, the smallest hydrodynamic size (151.2 ± 0.24 nm), the lowest polydispersity index (0.326 ± 0.04), and the highest ζ potential -25.6 ± 0.04 mV) compared to other formulations. These NPs effectively trapped C.O. (77.5 ± 0.44%) and released it gradually (89 ± 0.98%) over 72 h. Both C.O. and the C.O.-loaded NPs displayed significant antiviral activity against HSV-1. To understand this effect, we built a network analysis of HSV-1 genes and identified potential interactions between C.O. components and these genes. The top 10 hub genes, AKT1, TNF, EGFR, STAT3, SRC, BCL2, IL1B, HSP90AA1, PPARG, and MTOR, are reported. Additionally, computer modeling predicted how the chemical compounds of C.O. might interact with a key HSV-1 enzyme. These findings suggest that C.O./PLGA-PCL NPs hold promise as a new treatment for HSV-1 infections.

## Introduction

Herpes simplex virus (HSV) causes a common infection that can lead to painful blisters or ulcers. Through skin-to-skin contact, it spreads and is treatable but not curable. Numerous natural products have demonstrated their effectiveness in combating various viral diseases^1–3^.

Tropical and subtropical areas favor *Corchorus olitorius*, an annual plant with slender stems; it belongs to the Malvaceae family, and is used as a popular leafy vegetable^4^. It is a traditional dish that is widely consumed in the Middle East and various regions of Asia and Africa. Additionally, it possesses therapeutic properties^5^. It is considered as a nutrient-dense vegetable because of its high concentration of vitamins, minerals, and phenolic compounds^6^. As well as these names, it is also referred to as bush okra, wild okra, Jew’s mallow, meloukia, moroheia, moroheiya, mulu khiyah (and various spelling variants), nalta jute, and tasso^7^. *C. olitorius* is a stiff and fibrous annual herb that grows up to 4 m tall^7^. Moreover, *Corchorus olitorius* is also used as a herbal medicine against various diseases^8^. The infusion of leaves is used in some parts of Nigeria to treat female infertility, dysuria, ascites, and chest pain. Deficiencies in folic acid and iron are also treated with it^9–11^. The leaves are commonly used as a natural therapy for typhoid and malaria fevers^11^. Leaf twigs are used to cure heart disorders, while leaf infusion is used to stimulate appetite. Tanzanians utilize the leaves to relieve constipation^10,12,13^. Additionally, leaves are also used in Benin as a diuretic, emollient, and to alleviate malnutrition in infants^8^. Eleven phenolic acids in *C. olitorius* have been identified to have a range of biological activity. The potential antidiabetic and antihypertensive properties of plant may stem from its phenolic phytochemicals, which include caffeic acid and chlorogenic acid^14^. Apart from its neuroprotective characteristics, 3,4-caffeoylquinic acid demonstrated potent antioxidative attributes^15^. Terpenoids are extensively distributed in *C. olitorius*; 18 terpenoid substances have as yet been found. Diterpenoidal phytol compounds show antitumor, antibacterial, and antioxidant characteristics^16^. After being isolated from *C. olitorius*, corchoiononsides A, B, and C showed antinociception (high analgesia)^17,18^ besides having an antiallergic effect, are members of the ionone chemical class^19^. In *C. olitorius*, 14 flavonoidal derivatives have been found, including luteolin, kaempferol, and quercetin. Numerous biological activities have been revealed to be exerted by flavonoids^20^. They are widely recognized for their antioxidant and anti-inflammatory qualities and for supporting the cardiovascular and neurological systems^20^. Moreover, β-sitosterol and cardiac glycosides were among the steroidal compounds found in *C. olitorius*^19^. Due to its many advantages over alternative polymeric systems, poly(ε-caprolactone) (PCL) is a synthetic polymer that is often used in drug administration. Its semicrystalline structure gives it a longer half-life in the bloodstream and makes it biodegradable and biocompatible^21^. Furthermore, PCL degrades gradually, releasing medication over time, and is thought to be somewhat safe because it doesn not produce any acidic byproducts during this process. However, there are drawbacks to using PCL alone for drug administration, such as its high hydrophobicity, unstable aqueous solution, and quick reticuloendothelial system clearance^24^. To get around these restrictions, PCL is frequently altered with hydrophilic polymers, such as polylactide-co-glycolide (PLGA). Many synthetic and natural medications are delivered using PLGA, another biocompatible and biodegradable synthetic polymer. Instead of creating a copolymer, a physical mix of PCL and PLGA was created to integrate the unique benefits of both polymers. Whereas PLGA improves hydrophilicity and accelerates degradation, PCL provides prolonged release and delayed decay. Without the hassles of chemical production, a physical mix enables the best possible use of these qualities. Phase behavior studies were carried out to make sure that the mix was stable and miscible, and the results were adequate for the intended usage.

It has benefits including increased bioavailability, full and quick drug release via ester linkage hydrolysis, and safety. Here, the advantages of both polymers are combined to create nanocarriers that allow for regulated drug release: PLGA and PCL^23^ Despite its promising bioactive qualities, *Corchorus olitorius* fixed oil is poorly soluble, unstable under aqueous conditions, and has a limited bioavailability. These constraints impede its successful use in medication delivery. Encapsulation in PLGA-PCL nanoparticles addresses these concerns by improving the hydrophilicity of the oil, increasing its stability, and enabling controlled release. Therefore, the hydrophobic property of essential oils often makes them ineffective in therapeutic applications, resulting in low absorption and quick removal from the body. Several technologies, including microencapsulation, have been investigated to improve the characteristics of these oils. However, issues like instability in aquatic conditions and the absence of controlled release persist. This study provides a novel approach by encapsulating *Corchorus olitorius* fixed oil in PLGA-PCL nanoparticles, which not only increases the stability of the oil but also allows regulated and prolonged release. The purpose of this work was to generate PLGA-PCL NPs loaded with *C. olitorius* (also called C.O./PLGA-PCL-NPs) and investigate their potential effects against the *Herpes simplex* type 1 (HSV-1) virus. A chemical characterization of the fixed oil of *C. olitorius* was also conducted using gas chromatography coupled to mass spectrometry (GC-MS). Utilizing Design Expert® software, the emulsion (O/W) solvent evaporation process was used to produce *C. olitorius*/PLGA-PCL NPs utilizing PLGA-PCL mixes at 16 formulas. The formulation that exhibited the highest level of effectiveness in terms of hydrodynamic size, polydispersity index (PDI), ζ potential, and entrapment efficiency (EE%) was subjected to additional examination in terms of its shape, chemical composition, and percentage of release. Furthermore, the effect of *C. olitorius* and *C. olitorius*/PLGA-PCL NPs on the treatment of the HSV-1 virus indicates their possible use as an antiviral medication. Additionally, we built protein-protein interaction (PPI) networks using Cytoscape 3.10.1 software. Then we used the Cytohubba plug-in to select and extract the top 10 important genes based on their connection within the network. We also performed an in silico molecular docking investigation using all the components that were found, screening them against HSV-1 DNA polymerase (PDB ID: 2GV9) and HSV-1 complexed 5-iododeoxyuridine thymidine kinase (HSV-1 TK; PDB ID: 1KI7).

## Materials and methods

### Materials

#### Plant material

In February 2021, *Corchorus olitorius* seeds were acquired in the Minia Governorate of Egypt. Seed samples were kindly authenticated by Dr. Abd El-Halim A. Mohammed (Horticultural Research Institute, Department of Flora, and Phyto-taxonomy Research, Dokki, Cairo, Egypt). A voucher specimen **(Corc-2-2021)** was kept at the Department of Pharmacognosy, Faculty of Pharmacy, Deraya University, Egypt.

#### Chemicals

PCL (molecular weight 40,000) and poly(D,L-lactide-co-glycolide) (PLGA; 50:50) were acquired from Sigma Aldrich (Germany). Techno supplied polyvinyl alcohol (PVA; 98% hydrolyzed, MW ∼ 13,000), dichloromethane (DCM), and dimethylformamide. Analytical reagent grade was utilized for all other compounds, reagents, and solvents. The supplier of Vero cells was the Vacsera Research Foundation (Agouza, Giza, Egypt).

### Methods

#### Fixed oil extraction and separation from seeds of *Corchorus olitorius*

About 500 g of dry seeds of *Corchorus olitorius* were weighed and crushed using a domestic blender. *C. olitorius* seed powder was extracted using a continuous hot extraction process with a Soxhlet apparatus at a temperature of 40 °C for 4 h using petroleum ether as the solvent (1500 mL). The surplus solvent from the extract was removed using a rotary vacuum evaporator (Buchi Rotavapor R-300, Cole-Parmer, Vernon Hills, USA) under reduced temperature and pressure to produce the fixed seed oil. The oil was put into a little glass bottle, weighed to determine the percentage yield using dry seed powder, and kept at 18 °C until it was needed for further investigations.

#### Experimental design and selection of the best nanoparticle formula

To determine the best formulation for the nanoparticles, we utilized Design Expert® software version 10 to conduct a comprehensive investigation. This involved creating an optimal design to explore the impact of different factors on the PLGA-PCL-NPs formulation. The design encompassed four runs, with three independent variables and several dependent variables. The chosen formula was determined using a desirability function, which allowed us to consider all variables simultaneously. The formula with the most desirable characteristics, including the smallest particle size and polydispersity index, as well as the highest entrapment efficiency and zeta potential, was selected. This selection was made based on its high value of high desirability, indicating its closeness to the ideal solution. Subsequently, the chosen formula underwent further detailed characterization^25^.

#### Preparation of PLGA−PCL NPs loaded with *Corchorus olitorius* oil

**Figure 1.**
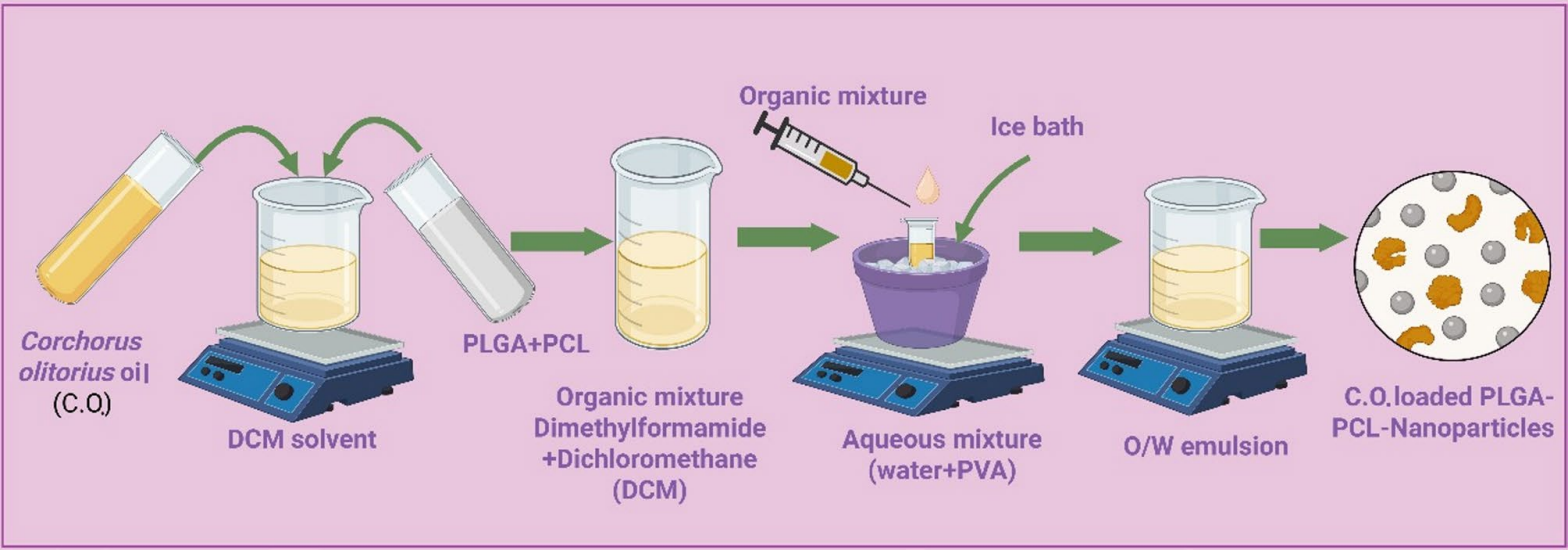
Graphic representation of the technique employed for the preparation of C*orchorus olitorius* oil-loaded PLGA/PCL-NPs by using emulsion (O/W) solvent evaporation.

Using PLGA and PCL in varying concentrations, an emulsion (O/W) solvent evaporation process was used to build nanoparticles (Table 1). Initially, 3 mL DCM were used to dissolve PCL, PLGA, and *C. olitorius* oil at room temperature. Subsequently, the organic mixture was gradually mixed with 30 mL of a 2% PVA water-based solution the resulting while being quickly agitated at 10,000 rpm for 10 min in an ice bath. The oil in water emulsion was then shaken for 5 h at room temperature using a magnetic stirrer to let the solvent drain and the particles solidify, as shown in Fig. 1. For use in the next tests, the desiccator holding the dried NP extract was kept at room temperature^26^.

**Table 1.** Optimal design for the optimization of *Corchorus olitorius* oil-loaded PLGA/PCL-NPs.

**Factors** **(independent variables)**	**Levels**		
	(low) −1	(medium) 0	(high) 1
X1: Volume of oil (mL)	0.3	0.6	1.2
X2: PLGA (mg)	14	16	18
X3: PCL (mg)	12	14	80
**Response (dependent variable)**	**Desirability constraints**		
Y1: Entrapment efficiency (EE%)	Maximize		
Y2: Size of vesicles	Minimize		
Y3: Zeta potential (ZP)	Maximize		
Y4: Polydispersity Index (PDI)	Minimize

#### In vitro characterization of PLGA−PCL NPs loaded with *C. olitorius* oil

##### Determination of nanoparticle size, polydispersity index (PdI), physical stability, and size distribution

The average particle size (PS), zeta potential (ZP), and polydispersity index (PDI) of PLGA-PCL nanoparticles were calculated at 25 °C using the dynamic light scattering technique and Zetasizer Nano ZS (Malvern Instruments, UK). To guarantee transparent dispersions with the right amount of scattering intensity, all formulations were suitably diluted before the experiments. Zeta potential and polydispersity index measurements were used to assess the physical stability of the formulations and the distribution of particle sizes, respectively. Each determination was performed three times to ensure accuracy.

##### *Corchorus olitorius* oil-entrapment efficiency of PLGA-PCL nanoparticles

The HEA10050, Gusto® high-speed, Heathrow centrifuge was used to separate the PLGA-PCL nanoparticles. After 15 min. of centrifuging the mixture at 9800× g, the liquid portion was diluted in ethanol for examination. Using a calibration curve and a UV–VIS spectrophotometer set at 310 nm, the quantity of *Corchorus olitorius* oil was determined. Next, using the below-shown formula (eq 1), the proportion of *C. olitorius* oils entrapment in the PLGA-PCL nanoparticles was determined:

1 $$\text{EE\%}=\frac{Ct-Cf}{Cf} \, \text{*} \, {100}$$

In the equation, Ct refers to the total amount of *Corchorus olitorius* in a 5 mL nanoparticle formulation, and Cf represents the amount of *Corchorus olitorius* that is free and has been dialyzed. These experiments were conducted three times for accuracy.

##### Selection of the optimum formula

For numerical optimization, we used Design-Expert® software version 10 from Stat-Ease Inc. (Minneapolis, Minnesota, USA). This procedure entailed determining which aspects were most crucial and disregarding the others that had little bearing. According to Table 2, we chose the formula with the highest EE% and absolute ZP value, as well as the lowest PS and PDI. For additional evaluation, we also employed the most ideal formula, aiming at a number around 1^27^.

**Table 2.** The independent variables levels used to formulate C.O.-loaded PLGA-PCL NPs.

**Formula** **No.**	**Independent**			**Response**			
	**X1:** **volume of oil (mL)**	**X2:** **amount of PLGA**	**X3:** **amount of PCL**	**Y1: entrapment efficiency (EE%)**	**Y2:** **size of vesicles**	**Y3:** **zeta potential (ZP)**	**Y4: polydispersity index** **(PDI)**
F1	0.4	16	16	56.8 ± 0.63	569.4 ± 0.24	−17 ± 0.02	0.574 ± 0.21
F2	0.4	16	14	59.8 ± 0.41	502.4 ± 0.61	−17.1 ± 0.03	0.451 ± 0.22
F3	0.2	14	16	30.2 ± 0.36	781.2 ± 0.51	−13.5 ± 0.05	0.514 ± 0.14
F4	0.6	14	14	59.8 ± 0.41	502.4 ± 0.02	−18 ± 0.02	0.451 ± 0.20
F5	0.2	16	14	51.3 ± 0.61	487.4 ± 0.41	−19.1 ± 0.03	0.501 ± 0.01
F6	0.2	18	12	41.8 ± 0.25	612.2 ± 0.31	−18 ± 0.01	0.521 ± 0.21
F7	0.4	16	14	63.8 ± 0.54	780.2 ± 0.42	−13.6 ± 0.01	0.489 ± 0.01
**MCO-F8**	**0.6**	**18**	**16**	**77.5 ± 0.44**	**151.2 ± 0.24**	**−25.6 ± 0.04**	**0.326 ± 0.04**
F9	0.2	18	16	49.1 ± 0.36	541.4 ± 0.34	−18.7 ± 0.03	0.514 ± 0.41
F10	0.4	18	12	58.4 ± 0.41	841.2 ± 0.41	−23.3 ± 0.0.12	0.394 ± 0.07
F11	0.4	18	14	42.3 ± 0.35	521.3 ± 0.81	−14.5 ± 0.02	0.53 ± 0.05
F12	0.2	16	14	51.3 ± 0.41	487.4 ± 0.14	−19.1 ± 0.14	0.501 ± 0.05
F13	0.4	16	16	59.4 ± 0.24	364.1 ± 0.61	−20.1 ± 0.02	0.418 ± 0.04
F14	0.6	16	12	48.4 ± 0.42	604.1 ± 0.36	−19.1 ± 0.04	0.591 ± 0.15
F15	0.4	14	12	55.8 ± 0.35	748.2 ± 0.41	−22.4 ± 0.04	0.514 ± 0.04
F16	0.2	14	12	36.5 ± 0.21	781.5 ± 0.54	−22.4 ± 0.21	0.514 ± 0.04

##### In-vitro characterization of the optimized formula (F8)

###### Morphological study by transmission electron microscopy (TEM)

To observe the PLGA-PCL nanoparticles of *Corchorus olitorius* oil, we used a transmission electron microscope, specifically the Hitachi H-7500 from Tokyo, Japan. For sample preparation, we spread 5 µL of the nanoparticles onto a 3 mm copper grid coated with carbon and let it sit for 10 min. Any excess sample was removed using filter paper, and then we applied 2 µL of a 2.5% uranyl acetate solution onto the sample grid for 2 min. The stained nanoparticles were then placed in a drying oven for 12 h. Finally, we operated the TEM at 100 kV to capture the sample image^29^.

###### Drug polymer interaction using Fourier transform infrared (FT-IR) spectroscopy

FTIR spectroscopy was used to investigate the interactions between the medication and the polymer using a Shimadzu 43,000 spectrophotometer from Kyoto, Japan. To do this, compact discs containing KBr were produced from samples of PLGA-PCL nanoparticles loaded with *Corchorus olitorius* oil. After that, the spectra were captured between 4000 and 400 cm^−1^, allowing for an average of 32 scans at a resolution of 2 cm^−1^^30^.

##### In vitro release studies

The dialysis bag method was used to measure the *in vitro* release rate of *C. olitorius* from MCO-F8 to mimic the circumstances of the gastrointestinal tract (GIT) in the human body. To clarify, a dialysis bag containing a determined amount of NPs with a cutoff molecular weight of 12-14 kD was used. The dialysis bag was then placed into a Jeio Tech SI-300, Seoul, Korea, shaking incubator that was set to 37 °C and spinning at 300 rpm. The 25 mL of phosphate-buffered saline (PBS, pH 5.5, 7.4) included 1.5% Tween 80 and 0.5% FBS. A 1-mL sample was taken at predetermined intervals and quickly replaced with an equivalent volume of heated buffer solution^31,32^.

A FLUOstar Omega microplate reader was used to measure the amount of *C. olitorius* that was liberated in the extracted sample at 310 nm^23^. The following formula (equation 2) was utilized to determine the release proportion (%):

2 $$\text{Release \%}=\frac{amount of released C.O.}{initial amount of loaded C.O.} \, \text{*} \, {100}$$

This analysis allowed for the consistent measurement of the amount of *C. olitorius* released over time, providing valuable insights into the release kinetics of *C. olitorius* from the NPs^22^.

### Antiviral activity

An MTT test was used to assess antiviral activity against the *Herpes simplex* type 1 (HSV-1) virus^32,33^.

#### Determination of the cytotoxicity of the samples on Vero cells

After a confluent sheet of Vero cells had developed, different concentrations from the tested samples were generated, and the cell monolayer was twice rinsed with wash media. The growth medium was then decanted from 96-well microtiter plates. The examined material was divided into twofold dilutions using DMEM. Each dilution was tested in 0.1 mL increments in several wells, with three wells serving as controls and receiving just maintenance media. For a maximum of 2 days, plates were incubated at 37 °C and constantly checked. The concentrations tested ranged from 3.9 to 62.5 µg/mL, physical indicators of toxicity, such as partial or total loss of the monolayer, rounding, shrinkage, or granulation of the cells, were examined in the cells. 20 µL of the MTT solution (BIO BASIC CANADA INC.) was added to each well after the MTT solution (5 mg/mL in PBS) was produced. The MTT was then completely mixed into the medium by placing it on a shaking table and shaking it for 5 min at 150 rpm. MTT was then allowed to metabolize for 4 h at 37 °C with 5% CO_2_. The medium was emptied off (if needed, the plate was dried with paper towels to remove residue). To completely combine the formazan and solvent, 200 µL of DMSO was used to resuspension formazan (MTT metabolic product). The shaker table was then set to 150 rpm for 5 min. At 560 nm, the optical density was measured, and at 620 nm, the background was eliminated.

There should be a direct relationship between cell amount and optical density. The maximum non-toxic concentration (MNTC) of each extract was calculated and used in further antiviral research.

#### MTT assay protocol

10,000 cells were plated in 200 µL of media per well in a 96-well plate using the MTT assay. Three empty wells were designated for the blank controls. After that, the wells were incubated for a whole night at 37 °C with 5% CO_2_ to encourage the cells to stick to them. Following that, the wells were shaken at 150 rpm for 5 min while being incubated for 1 h at a volume equivalent to a nonlethal dilution of the virus suspension and the tested sample. Additionally, 100 µL of the prepared treatment mixture (virus + nanoparticles) was added to each well was added to the viral/sample suspension.

To allow the virus to replicate, the sample and virus were incubated for 1 d at 37 ºC with 5% CO_2_. Triplicates were performed using the same concentration range (3.9–62.5 µg/mL). After adding 20 µL of MTT solution to each well and shaking the 96-well plates for 5 min at 150 rpm, the MTT was well mixed into the medium. In PBS, a minimum of 2 mL of MTT solution with a 5 mg/mL concentration was made. After that, the plate was incubated at 37 °C with 5% CO_2_ for 1-5 h to allow the MTT to metabolize. After removing the media, the plate was dried out using paper towels if necessary to get rid of any last bits of residue. The formazan (MTT metabolic product) was re-suspended in 200 µL of DMSO and shaken at 150 rpm for 5 min on a shaking table to thoroughly mix it into the solvent. The optical density was determined at 560 nm, and the background was removed at 620 nm. An exact correlation between the number of cells and the optical density was taken.

### GC/MS analysis of the isolated *Corchorus olitorius* oil

A trace GC-TSQ mass spectrometer (Thermo Scientific, Austin, TX, USA) with a direct capillary column TG–5MS (30 m x 0.25 mm x 0.25 µm film thickness) was used to analyze the chemical composition of the samples. The temperature of the oven started at 50 °C and was increased by 50 °C per minute for 4 min. until it reached 250 °C. It was then elevated to a final temperature of 300 °C over the next 2 min, rising by 25 °C per min. The MS transfer line and injector were kept at 260 and 270 °C, respectively. Helium was used as the carrier gas, with a constant flow rate of 1 mL/min. With a 4-minute solvent delay, diluted samples with 1 µL were automatically injected using an Autosampler AS1300 coupled to a GC in split mode. In full scan mode, electron ionization (EI) mass spectra were recorded at 70 eV across the *m/z* range from 50 to 650. An ion source temperature of 200 °C was selected. The components were identified by comparing their mass spectra with those found in the NIST 14 and WILEY 09 mass spectral databases^35^.

### Network pharmacology-based analysis

#### Screening of *Corchorus olitorius* extract-related target genes

The Traditional Chinese Medicine Systems Pharmacology Database and Analysis Platform (TCMSP) database was searched to identify target genes for compounds present in *Corchorus olitorius* extract.

These findings were derived from protein interactions, pharmacophore models, and chemical similarities (https://old.tcmsp-e.com/index.php)^36^, from the Comparative Toxicogenomics Database (CTD) (http://ctdbase.org/) and the SwissTargetPrediction database (http://www.swisstargetprediction.ch/). These target genes were then converted into their canonical gene names using the UniProt database (https://www.uniprot.org/)^37^.

#### Screening of Herpes simplex-related target genes

Genes linked to infection with the HSV-1 virus were gathered from the Comparative Toxicogenomics Database CTD (https://ctdbase.org/), DisGeNET database (https://www.disgenet.org/), and Gene cards (https://www.genecards.org/) using the keywords"HSV-1 virus", also known as the Herpes simplex virus, and the species restricted to “Homo sapiens”. Using Venny 2.1, duplicate targets were eliminated, and overlapping component- and disease-related proteins were found (https://bioinfogp.cnb.csic.es/tools/venny/)^38^ intersections as potential targets of these components in Herpes simplex virus infection.

#### Protein-protein interaction (PPI) network construction

The STRING version 12.0 on a PPI network (https://string-db.org/)^39^ was created using a target gene query list and exported to the free molecular and genetic interaction network visualization, modeling, and analysis program Cytoscape 3.10.1 (USA)^39^. The top 10 significant genes were then filtered using the Cytohubba plug-in.

### In silico molecular docking study

The previously outlined procedure was followed in carrying out the molecular docking investigations^40^ and processed using the AutoDock VINA software (version 0.8, The Scripps Research Institute, La Jolla, CA, USA) combined with a PyRx virtual screening tool. To further visually represent the docking data, the Discovery Studio visualizer version v19.1.0.18287 (BIOVIA, San Diego, CA, USA) was utilized. The RCSB Protein Data Bank provided the three-dimensional (3D) crystal structures of HSV-1 DNA polymerase (PDB ID: 2GV9) and thymidine kinase from HSV-1 complexed with 5-iododeoxyuridine (HSV-1 TK; PDB ID: 1KI7) (www.rcsb.org). 5-Iododeoxyuridine and guanidine were extracted from (PDB ID: 1KI7) and (PDB ID: 2GV9), respectively, and re-docked back into their receptors to confirm the molecular docking results. The docking data, which are based on hydrogen bond, hydrophobic, and electrostatic interactions, were reported as binding energy values (kcal/mol) of ligand-receptor complexes. The necessary docking settings were generated as previously reported, including the creation of PDBQT files for the ligands and receptors, the identification of binding sites, the computation of the protonation state, and the overall charges^41^.

## Results and discussion

### Statistical analysis and formulation optimization

With the help of the Design Expert® software of Stat-Ease Inc. (Minneapolis, Minnesota, USA), version 10.0.0.0, we developed nanoparticles using surface response optimum statistical design. The objective of this method was to achieve an ideal formulation of nanoparticles, as indicated in Table 1, with reduced PS and PDI values and increased EE% and ZP values. We looked at the individual and interacting impacts of the independent variables within this framework; they are listed in Table 2. We developed polynomial equations for each answer (Y1, Y2, Y3, and Y4) using regression analysis. An effect was said to be positive if the regression coefficient was positive; the converse was stated if the sign was negative^42^. In addition, we used the ANOVA test to determine significance at a *p*-value of less than 0.05. Furthermore, to illustrate the important impacts of independent variables on formulation answers and ascertain the ideal level for every variable, we employed three-dimensional (3D) surface plots.

### In vitro evaluation of PS, PDI, ZP, and EE% of PLGA-PCL-NPs loaded *Corchorus olitorius* oils formulas

**Figure 2.**
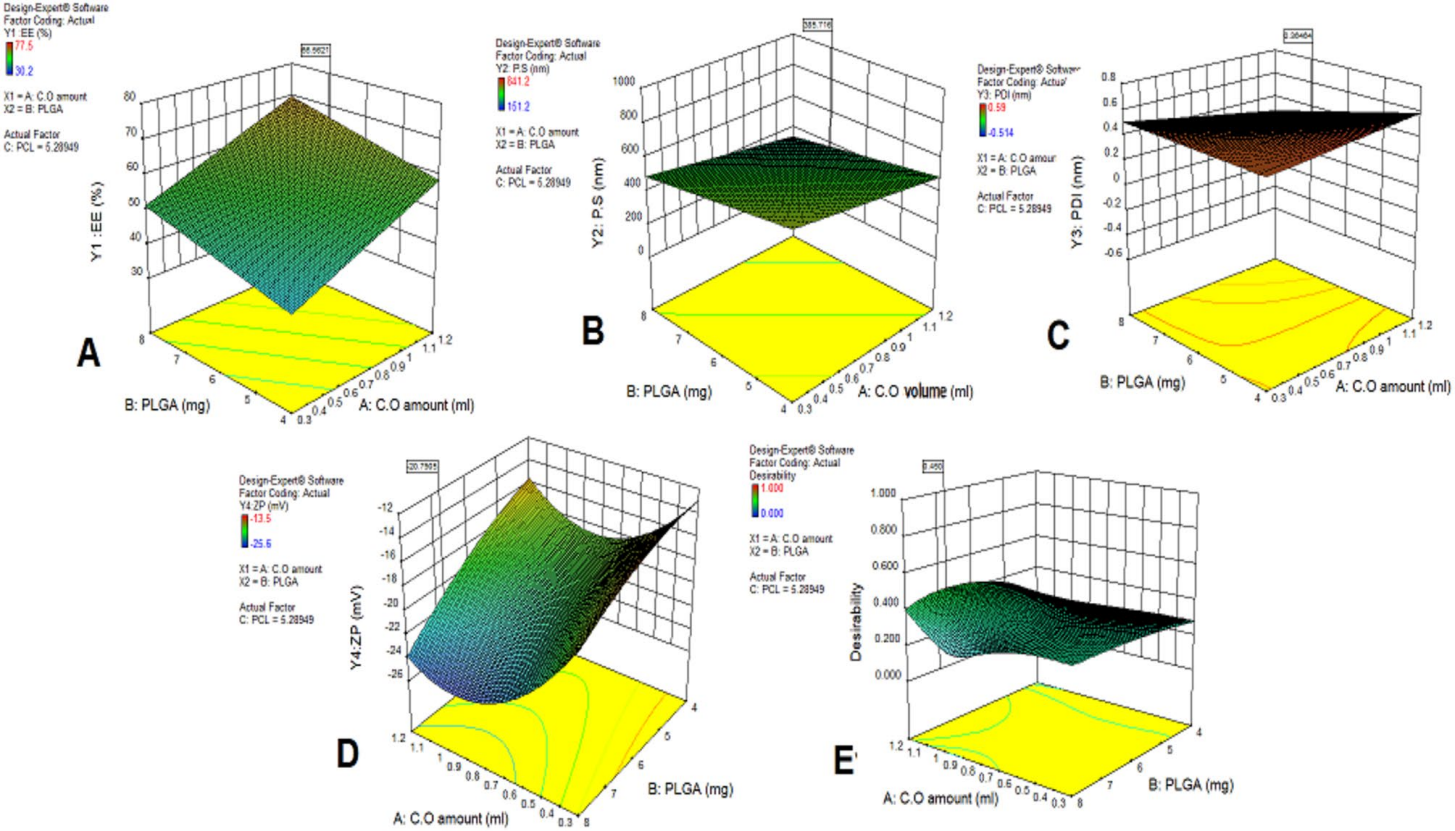
Response 3D plots for the effect of the Oil, PLGA, and PCL amounts on (**A**) EE%; (**B**) PS; (**C**) PDI; (**D**) ZP; and (**E**) desirability of PLGA-PCL nanoparticles. Abbreviations: *C. olitorius*: *Corchorus olitorius* oil, PLGA: Poly(D,L-lactide-co-glycolide), PCL: poly(ε-caprolactone), EE% - entrapment efficiency percentage, PS, particle size; PDI, polydispersity index; and ZP, zeta potential.

**Figure 3.**
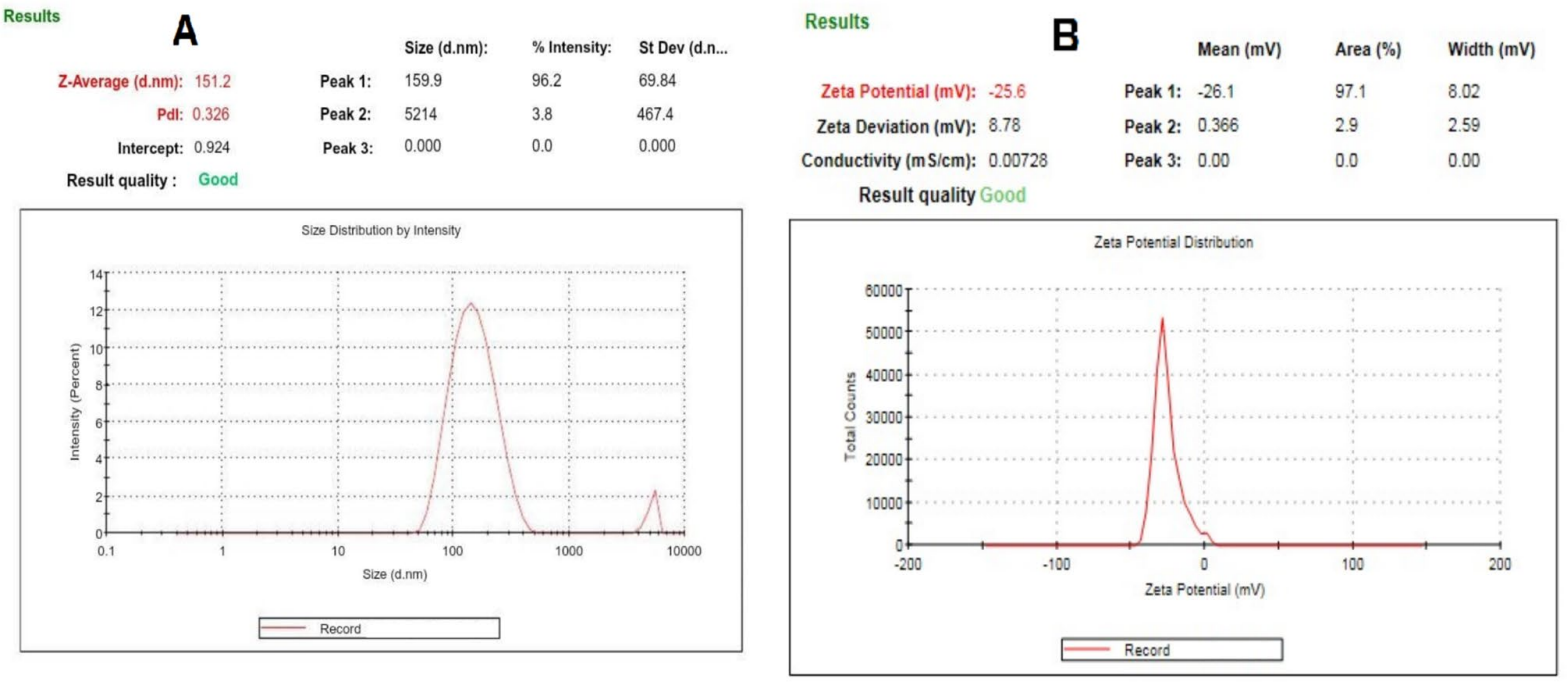
A size distribution report of MCO-F8, and B zeta potential report of MCO-F8.

The development of C.O./PLGA-PCL NPs involved the evaluation of several combinations of oils, PLGA, and PCL. PLGA-PCL nanoparticles were selected for encapsulating *Corchorus olitorius* fixed oil due to their excellent biodegradability, biocompatibility, and ability to control the release of hydrophobic compounds. The combination of the hydrophilic nature of PLGA and the slower degradation rate of PCL provides an ideal matrix for the sustained delivery of bioactive agents. After evaluating the hydrodynamic size, PDI, ζ potential, and EE%, the best formulation was selected for further research. The average hydrodynamic diameters, PDI, ζ potential, and EE% of the C.O./PLGA−PCL NP formulations made with varying concentrations of oils, PLGA, and PCL are shown in Table 2 and in Figs. 2 and 3. Notably, when compared to other formulations, the C.O.-PLGA−PCL NPs made with 0.6 mL of C.O. oils, 18 mg of PLGA, and 16 mg of PCL (MCO-F8 formula) had the lowest hydrodynamic size (151.2 ± 0.24 nm) and PDI (0.326 ± 0.04) as well as the greatest ζ potential (−25.6 ± 0.04 mV). The small size of F8 may contribute to the passive build-up of NPs in tissues with poor lymphatic drainage and permeable vasculature. Furthermore, a homogeneous dispersion of NPs was ensured by the low PDI value of the F8 formula, which showed a uniform and narrow particle size range. Moreover, the large negative surface charge of the F8 formula could inhibit NP aggregation and improve their long-term stability. Furthermore, in comparison to the other formulations, the F8 formulation showed a greater EE% (77.5 ± 0.44%), which may have been caused by its smaller particle size. Greater trapping of C.O. within the nanoparticles was made possible by the higher surface area-to-volume ratio of the F8 formula, which ultimately increased its therapeutic potential^43^. While PLGA alone can encapsulate hydrophobic substances, the addition of PCL was required to control the release rate of hydrophobic essential oils. The hydrophobic characteristic of PCL inhibits its release, ensuring that the oil is gradually released over time, which is critical for sustaining therapeutic effectiveness. The dual-polymer approach of combining PCL and PLGA was chosen to take advantage of the distinct properties of each polymer. PLGA enhances the hydrophilicity and faster degradation rate, while PCL provides a slower degradation rate and sustained release. This combination allows for a controlled release profile, which is essential for maintaining therapeutic efficacy over an extended period. The differences in mechanical properties and degradation rates of these polymers were carefully considered to align with the objectives of the study on controlled drug delivery. These results highlight the importance of the content of the formulation on the physical and chemical characteristics of the nanoparticles, indicating the possibility of effective and targeted administration of the active components, as shown in Table 2.

### In vitro characterization of the optimized formula (MCO-F8)

#### Morphological study by transmission electron microscopy (TEM)

**Figure 4.**
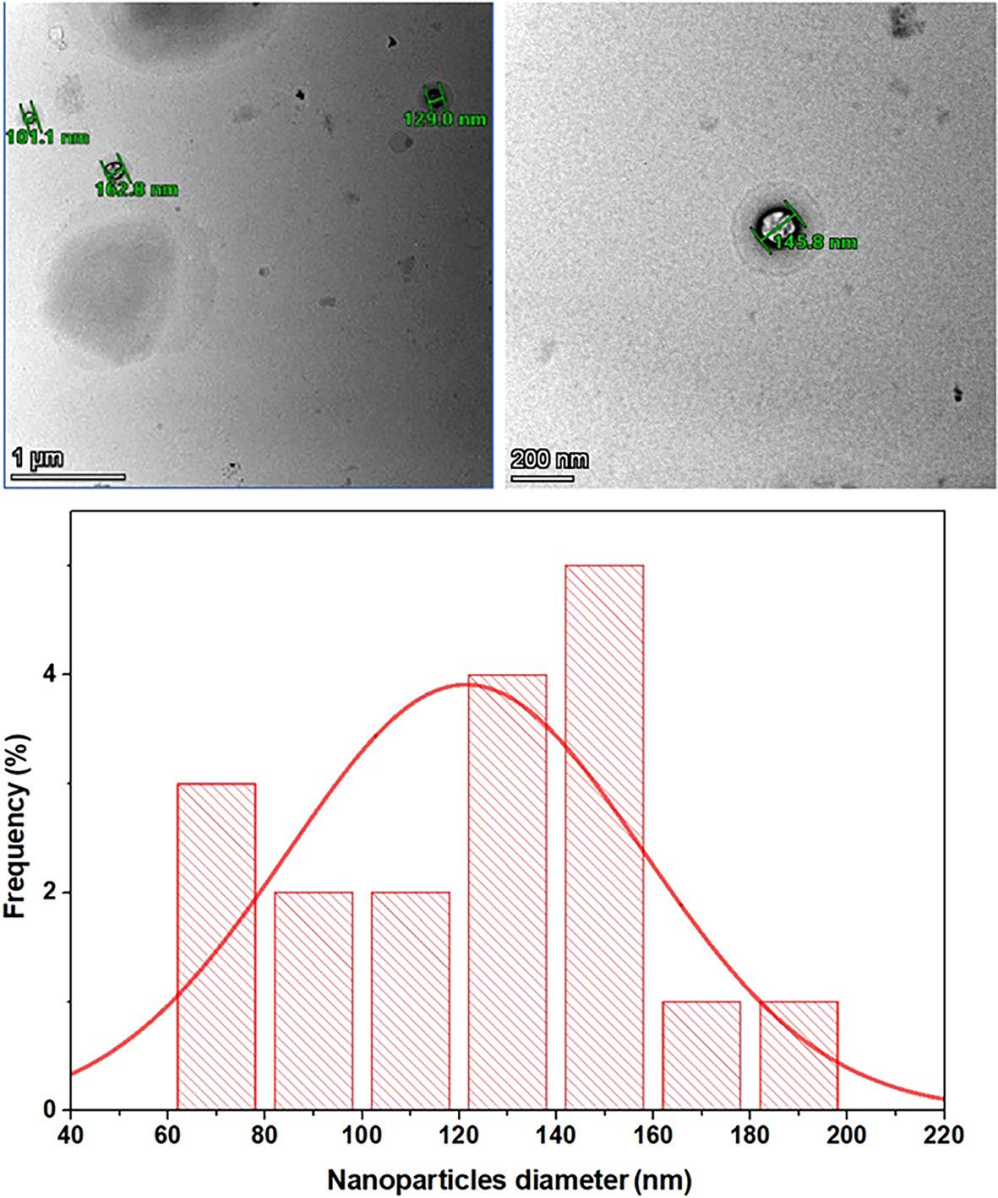
Transmission electron microscope (TEM) pictures of the optimal formula and the diameter histogram of MCO-F8.

TEM was utilized to examine the shape and size of the MCO-F8, produced with 0.6 mL C.O. oils, 18 mg of PLGA, and 16 mg of PCL. As illustrated in Figure 4, the NPs displayed a round shape without any apparent clustering. The absence of aggregation, on the other hand, can be attributed to the MCO-F8 strongly negative charge, as previously reported in the ZP data. Highly charged MCO-F8 repel each other, decreasing aggregation. MCO-F8 had an average diameter. The average size of the preferred MCO-F8 formula ranged from 60 nm to 200 nm, as determined by ImageJ at the National Institutes of Health in the USA. TEM was used to identify smaller particle sizes since the dynamic light scattering technique overestimates the particle size of nanoparticles due to Brownian motion.

#### Drug-polymer interaction using Fourier transform infrared (FT-IR) spectroscopy

**Figure 5.**
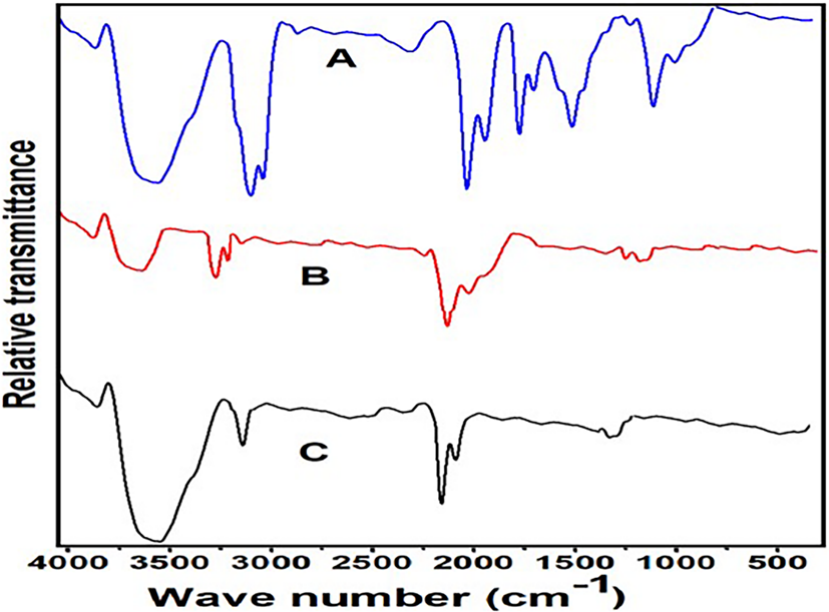
FTIR spectra of (**A**) *Corchorus olitorius* oil (BO); (**B**) blank PLGA-PCL NPs; and (**C**) MCO-F8.

To confirm the integrity of the physical blend, we performed a comprehensive characterization of the PCL-PLGA blend. FTIR analyses were carried out, revealing the absence of significant chemical interactions between the polymers. The results indicated that the blend maintains the distinct characteristics of both polymers without the formation of a copolymer. The FTIR spectra in Fig. 5 (**A-C**) give important evidence for the effective formation of *C. olitorius*-loaded PLGA-PCL nanoparticles. In Fig. 5A, the spectrum of *C. olitorius* extract alone shows three distinct peaks: a band at 2890 cm⁻^1^, corresponding to C−H stretching vibrations of aliphatic chains, another at 1500 cm⁻^1^, assigned to C−H bending, and a third peak at 1350 cm⁻^1^ indicating C−O stretching, likely originating from hydroxy and ester groups within the phytoconstituents of the extract. These findings are congruent with earlier research on plant-derived bioactives, which are rich in polyphenolic and ester-containing compounds. Figure 5B shows the FTIR spectra of blank PLGA-PCL nanoparticles. Four prominent peaks were observed: a broad band at 3305 cm⁻^1^ for O-H stretching, a peak at 2950 cm⁻^1^ for C-H bending of methylene groups, a strong signal at 1727 cm⁻^1^ for ester C=O stretching, and a band at 1094 cm⁻^1^ for C-O stretching. These peaks demonstrate the existence of PCL and PLGA components and are consistent with FTIR profiles described in previous research for these polymers. Notably, the intense carbonyl stretching band is characteristic of polyester-based biodegradable matrices. Figure 5C shows the spectrum of the final nanoparticle formulation that incorporates *C. olitorius* into the PLGA-PCL matrix. Here, the typical peaks of both the plant extract and the copolymer can be seen. The C=O and C-O peaks from PLGA-PCL remain well defined, whereas the C-H and C-O bands from *C. olitorius* can still be detected with minor shifts. The retention of these peaks, with no substantial chemical change or disappearance, indicates that *C. olitorius* was physically entrapped within the nanoparticles rather than experiencing covalent bonding or disintegration. This result is corroborated by comparative investigations in the literature, which show that physical encapsulation often preserves the different functional groups of both the carrier and the medication. The FTIR spectra demonstrates the effective encapsulation of *C. olitorius* into PLGA-PCL nanoparticles, demonstrating the integrity of both the polymeric carrier and the loaded phytochemicals. The absence of substantial shifts or new peak formation suggests a physical interaction rather than a chemical modification, which is consistent with earlier research on herbal-loaded polymeric systems.

#### In-vitro release

**Figure 6.**
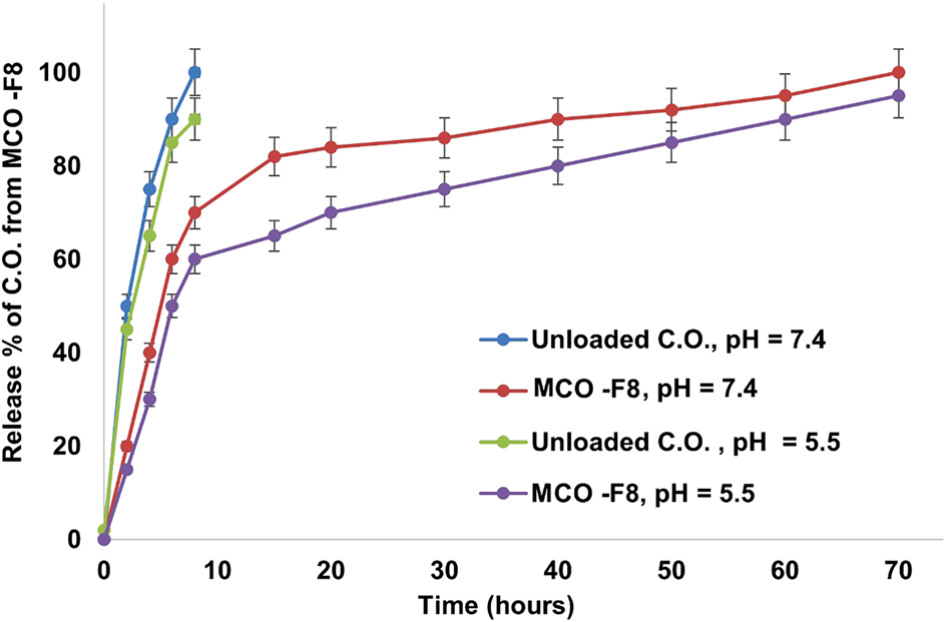
Time-dependent release % of unloaded C.O. and C.O. from C.O./PLGA−PCL NPs at 37 °C, into phosphate buffer (pH 7.4 and 5.5)**.**

The proportion of *C. olitorius* released into a 37 °C PBS medium from both the unloaded and the MCO-F8 strains is shown in Figure. 6. Notably, after under 8 h, C.O. from MCO-F8 shows almost full diffusion (89 ± 0.98%) off the dialysis membrane *C. olitorius*, on the other hand, exhibits continuous release behavior over 72 h; after 8 and 72 h at pH 7.4, respectively, (45.01 ± 1.025%) diffused from the dialysis bag.

*C*. *olitorius* exhibits a fast release behavior over 72 h at the pH of the extracellular fluid (pH 5.5), with 61.02 ± 1.05% and 94.05 ± 1.98% diffusing from the dialysis bag after 8 and 72 h, respectively. A regulated, prolonged release is achieved by combining PLGA with PCL, which strikes a balance between release rates. When PLGA is used, loaded *C. olitorius* diffuses quickly from the polymeric matrix due to polymer swelling upon PBS exposure, which erodes the polymeric matrix. Furthermore, the quick release of *C. olitorius* is facilitated by the breakage of the ester bond in the PLGA. On the other hand, because PCL is semicrystalline and hydrophobic, it causes sluggish diffusion rates out of the polymeric matrix, which causes the polymer to hydrolyze slowly and release the loaded medicine over an extended time. PLGA-PCL exhibits a larger release of fixed oil at pH 7.4 compared to pH 5.5, due to faster PLGA hydrolysis at neutral pH. PCL partially counters this, but cumulative release remained higher at pH 7.4

As a result, the PLGA-PCL polymer mix used in the construction of polymeric NPs produces moderate drug release rates.

### Antiviral activity against Herpes simplex type 1 (HSV-1)

To assess the cytotoxicity of nanoparticles on Vero cells, the MTT assay was employed to ensure that the quantities of *C. olitorius* fixed oil sample and the produced nanoparticles are non-toxic to Vero cells *C. olitorius* fixed oil has a maximum nontoxic concentration (MNTC) of 62.5 µg/mL, while its nanoparticles have an MNTC of 3.9 µg/mL.

**Figure 7.**
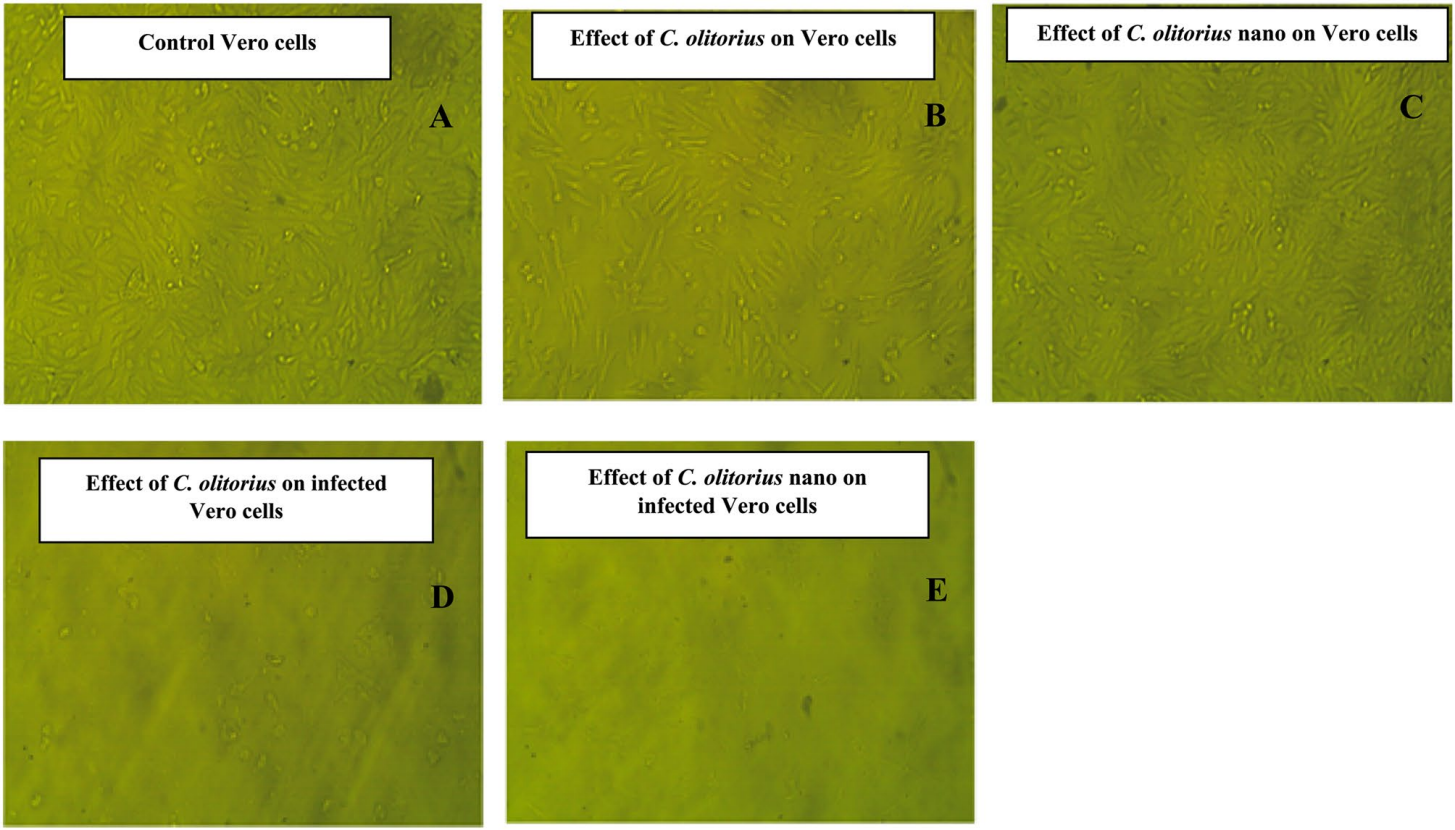
(**A**) Control Vero cell; (**B**) effect of *C. olitorius* on Vero cells (62.5 µg/mL); (**C**) effect of *C. olitorius* nano on Vero cells (3.9 µg/mL); (**D**) effect of *C. olitorius* on infected Vero cells (31.25 µg/mL); (**E**) effect of *C. olitorius* nano on infected Vero cells (1.95 µg/mL).

The antiviral and cytotoxic effects of the *C. olitorius* fixed oil and the nanoformulation on HSV-1 viruses were investigated using the MTT antiviral test technique. High levels of antiviral activity against the HSV-1 virus were demonstrated by the *C. olitorius* fixed oil, with an IC_50_ of 31.25 µg/mL and a nanoformulation of 1.95 µg/mL **(**Figure. 7**)**.

### GC/MS analysis of the isolated *Corchorus olitorius* oil

**Figure 8.**
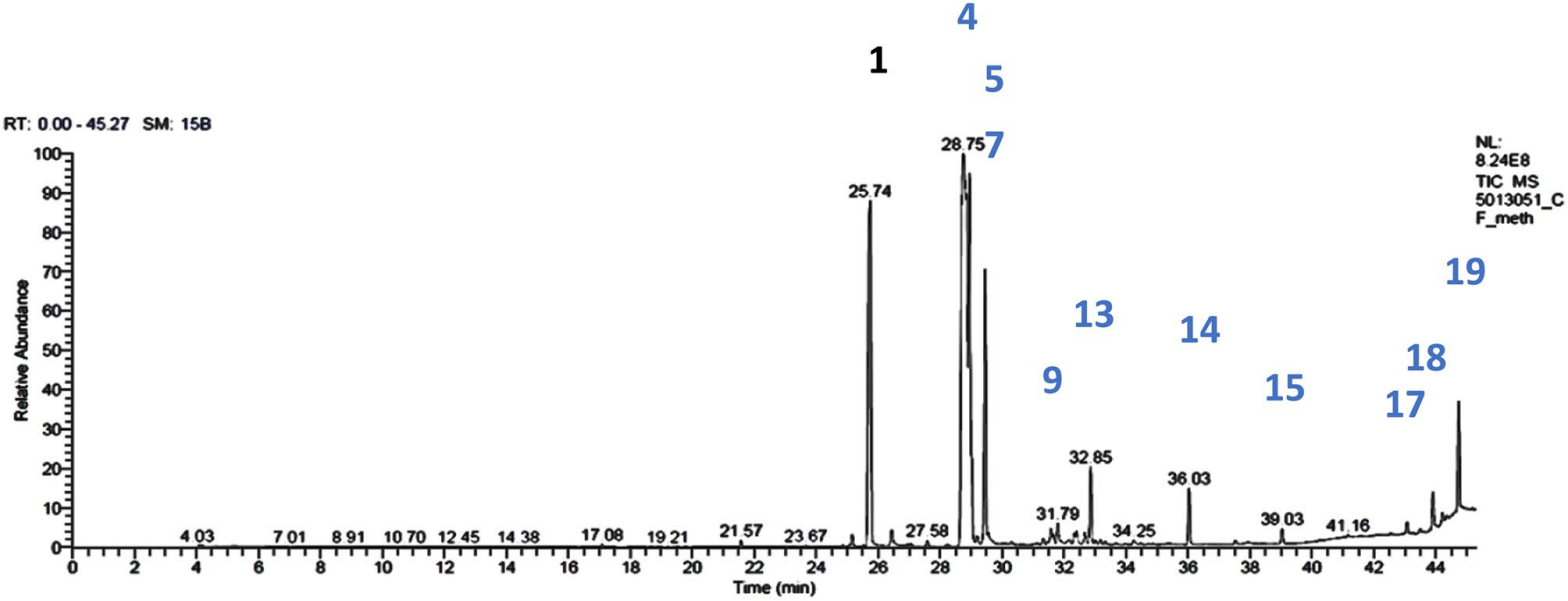
GC/MS analysis of the fixed oil extracted from *Corchorus olitorius* seeds.

**Figure 9.**
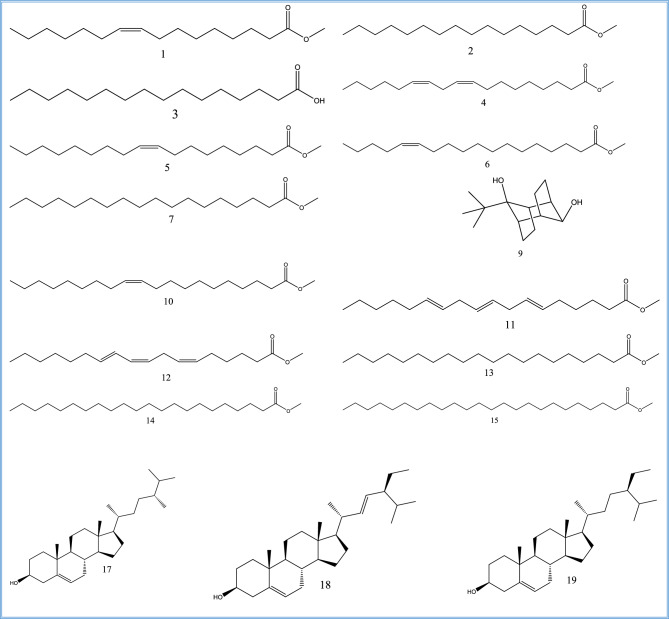
Compounds **1**-**7**, **9**-**15**, and **17**-**19**, identified from GC/MS analysis of the fixed oil extracted from *Corchorus olitorius* seeds.

In the current study, the fixed oil of *Corchorus olitorius* seeds was subjected to GC/MS technique to identify its constituents. By GC/MS analysis of *C. olitorius* fixed oil, 17compounds were identified (Figs. 8 and 9). Relative area percentages and retention times of the identified compounds are listed in Table 3. Fatty acids and sterols are the two main categories in the oil sample. The percentage of fatty acids identified (92.83%) was greater than the percentage of sterols (6.11%). Fatty acids were the major constituents characterized in the extracted oil, including linoleic acid methyl ester with a 38.87 relative area percentage; palmitic acid, methyl ester (21.39%); oleic acid, methyl ester (11.22%); and stearic acid methyl ester (10.76%). The percentage of saturated fatty acids is 38.47%, and the unsaturated fatty acid represents 54.36%. The only reported sterols are β-sitosterol (4.13%), campesterol (1.53%), and stigmasterol (0.45%).

**Table 3.** Results of GC/MS analysis for the fixed oil extracted from *Corchorus olitorius* seeds.

**No.**	**Identified compound**	**R**_**t**_	**RR**_**t**_	**B.P.**	**M**^**+**^	**Mol. formula**	**Area %**
1	9-Hexadecenoic acid, methyl ester, (*cis*)-	25.16	0.875	55	268	C_17_H_32_O_2_	0.47
2	Hexadecanoic acid, methyl ester (= palmitic acid, methyl ester)	25.74	0.895	74	270	C_17_H_34_O_2_	21.39
3	*n*-Hexadecanoic acid	26.43	0.919	73	256	C_16_H_32_O_2_	0.66
4	Linoleic acid methyl ester (= 9,12-octadecadienoic acid (*cis,cis*)-, methyl ester)	28.74	1	67	294	C_19_H_34_O_2_	38.87
5	9-Octadecenoic acid (*cis*)-, methyl ester (= oleic acid, methyl ester)	28.95	1.007	55	296	C_19_H_36_O_2_	11.22
6	*cis*−13-Octadecenoic acid, methyl ester	29.02	1.009	55	296	C_19_H_36_O_2_	1.57
7	Octadecanoic acid, methyl ester (= stearic acid methyl ester)	29.45	1.024	74	298	C_19_H_38_O_2_	10.76
8	Unidentified	31.57	1.098	-	-	-	0.56
9	9-*tert.*-Butyltricyclo[4.2.1.1(2,5)]decane-9,10-diol	31.79	1.106	149	224	C_14_H_24_O_2_	0.89
10	*ci*s-11-Eicosenoic acid, methyl ester	32.33	1.124	55	324	C_21_H_40_O_2_	0.38
11	6-*trans*,9-*trans*,12-*trans*-Octadecatrienoic acid, methyl ester	32.41	1.127	67	292	C_19_H_32_O_2_	0.46
12	Methyl 6-*cis*,9-*cis*,11-*trans*-octadecatrienoate	32.66	1.136	67	292	C_19_H_32_O_2_	0.50
13	Eicosanoic acid, methyl ester (= methyl eicosanoate)	32.85	1.143	74	326	C_21_H_42_O_2_	2.88
14	Docosanoic acid, methyl ester	36.03	1.253	74	354	C_23_H_46_O_2_	2.14
15	Tetracosanoic acid, methyl ester	39.03	1.358	74	382	C_25_H_50_O_2_	0.64
16	Unidentified	43.06	1.498	-	-	-	0.50
17	Campesterol	43.90	1.527	43	400	C_28_H_48_O	1.53
18	Stigmasterol	44.20	1.537	55	412	C_29_H_48_O	0.45
19	β-Sitosterol	44.73	1.556	107	414	C_29_H_50_O	4.13
**Percentage of identified compounds**						**98.94%**	
**Percentage of unidentified compounds**						**1.06%**	

R_t_ = Retention time; RR_t_ = retention time relative to "linoleic acid methyl ester"(R_t_ = 28.74); B.P. = base peak; M^+^ = molecular ion peak.

### Network pharmacology-based analysis

Due to the impressive antiviral effects of *Corchorus olitorius* oil against the HSV-1 virus, we acknowledge the critical importance of developing a gene network for Herpes simplex virus-1. This initiative is crucial for identifying potential drug targets within biological systems, and understanding the interactions between drugs, proteins, and pathways helps prioritize specific molecular targets for therapeutic intervention.

#### Screening of target genes related to *Corchorus olitorius* oil

A total of 460 targets were found to be associated with the compounds of ***C. olitorius* oil** from the TCMSP and SwissTargetPrediction. Using the UniProt database, these genes were renamed to their standard gene names.

#### Screening of genes related to the HSV-1 virus

1616 Herpes simplex-related targets were screened out from CTD, GeneCards, and DisGeNET databases using the search terms "Herpes simplex virus“or”HSV-1 virus"and limiting results to Homo sapiens. To compare the probable HSV-1 viral targets from each database with the targets regulated by compounds found in *C. olitorius*, a Venn diagram was made.

**Figure 10.**
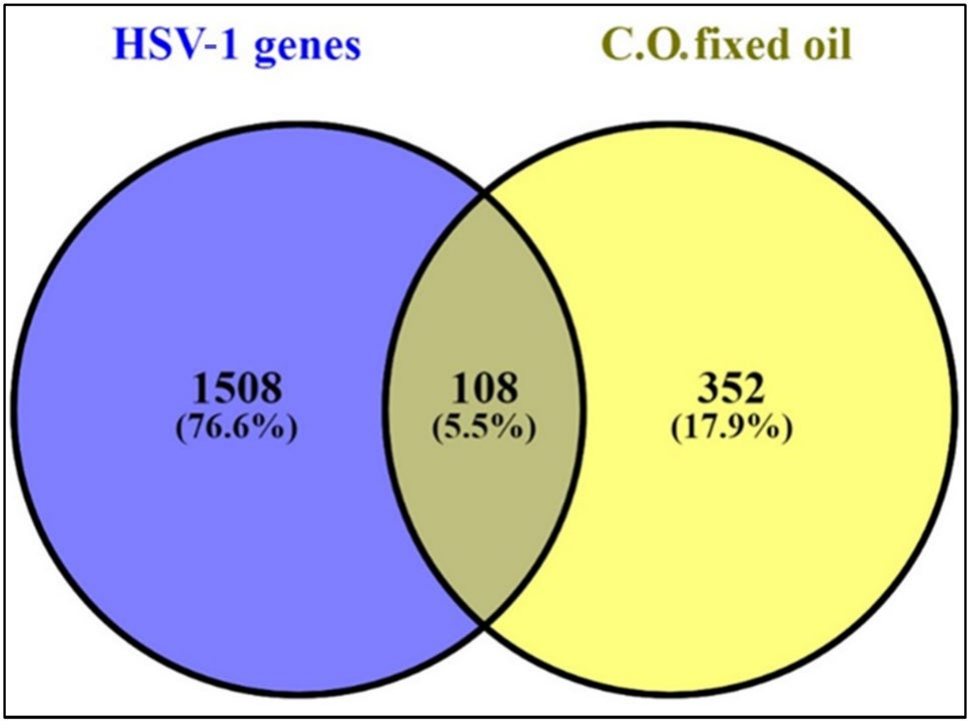
Venn diagram for the integrated analysis of the related targets of* C. olitorius* extracted compounds and **HSV-1 virus** targets.

This revealed a total of 108 genes after removing duplicates that were commonly intersected HSV-1 virus targets from three databases (Fig. 10).

#### Protein-protein interaction (PPI) network construction

**Figure 11.**
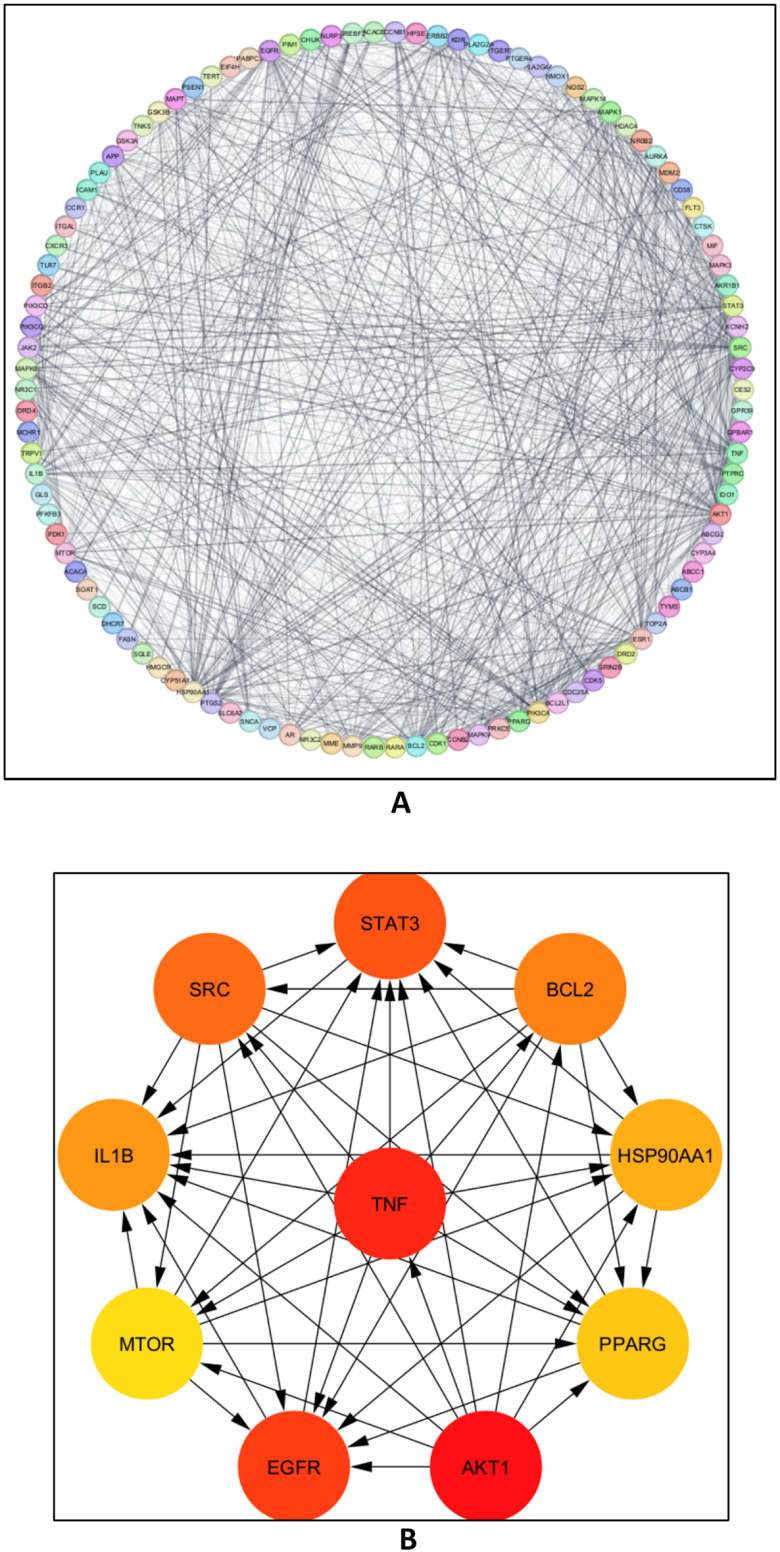
(**A**), network nodes stand in for 108 protein targets, and the edges show connections between proteins; in (**B**), network nodes stand in for the top 10 hub genes; the stronger the relationship and the higher the score, the darker the color.

The 108 overlapping targets from the aforementioned Venn database were imported into the STRING database to examine their interactions to get information on the protein-protein interaction network. Furthermore, as seen in Fig. 11A, the PPI network was built using Cytoscape 3.10.1 and has 106 nodes, 1274 edges, and an average node connectivity of 24.04. Based on their level of network connection, the top 10 most important genes were then found and extracted using the CytoHubba plug-in, as seen in Figure. 11B. Among these genes were AKT1, TNF, EGFR, STAT3, SRC, BCL2, IL1B, HSP90AA1, PPARG, and MTOR. Table 4 summarizes the topological characteristics, such as node degree, betweenness, and proximity, for each of these proteins.

**Table 4.** Topological parameters of the top 10 hub genes.

No.	Name	Target	Degree	Betweenness	Closeness
1	**RAC-α serine/threonine-protein kinase**	AKT1	78	0.0749	0.7955
2	**Tumor necrosis factor**	TNF	72	0.0672	0.7609
3	**Epidermal growth factor receptor**	EGFR	66	0.0532	0.7292
4	**Signal transducer and activator of transcription 3**	STAT3	65	0.0438	0.7143
5	**Proto-oncogene tyrosine-protein kinase**	SRC	64	0.0792	0.7047
6	**Apoptosis regulator**	BCL2	62	0.0263	0.7000
7	**Interleukin-1 β**	IL1B	61	0.0379	0.7000
8	**Heat shock protein HSP 90-α**	HSP90AA1	59	0.0503	0.6954
9	**Peroxisome proliferator-activated receptor γ**	PPARG	53	0.0287	0.6646
10	**Estrogen receptor-1**	ESR1	52	0.0166	0.6646

### Molecular docking study

Aiming for cellular and viral enzymes that play important roles in the HSV-1 replication cycle may aid in the creation of potent, broad-spectrum antiherpetic medications. For example, it has been found that HSV-1 DNA polymerase is a necessary enzyme for viral replication^41^, DNA polymerase uses dTMP as a substrate during viral DNA replication, even though HSV-1 TK is a crucial enzyme that catalyzes the transfer of the γ-phosphate group of ATP to thymidine in the salvage pathway of pyrimidine synthesis^44^. The binding pocket in 1KI7 is mostly made up of the conserved amino acids Tyr 101, Arg 163, Tyr 172, Gln 125, and Glu 225. H-bonds and the pi-pi interaction between ligands and amino acid residues are the two most important interactions, while the primary interactions of 2GV9 with Gly 385 and Asp 481 involve H-bonds.

**Figure 12:**
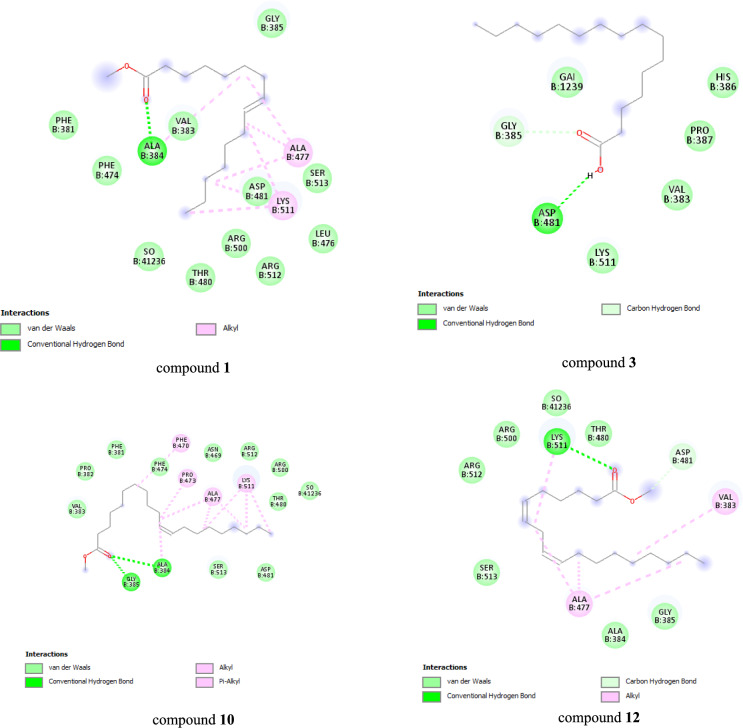
The interaction between the top-scoring compounds** 1**, **3**, **10**, and **12**, with the active sites of HSV-1 DNA polymerase.

The docking results, as shown in Fig. 12 demonstrated that compounds **1**, **3**, **10**, and **12** had a significant ability to bind appropriately to the active sites of HSV-1 DNA polymerase as the crystallized ligand, with detected binding energies of −8.0 kcal/mol. Conversely, none of the compounds interacted with the HSV-1 thymidine kinase (HSV-1 TK) active site, with detected binding energies of −3.3 kcal/mol.

The formation of many fundamental contacts between the interacting amino acid residues of the active sites of the target enzymes and the functional groups of compounds **1**, **3**, **10**, and **12** have established molecular interactions. In addition to van der Waals contacts, these interactions were found to be hydrogen bonds, carbon-hydrogen bonds, and hydrophobic (alkyl hydrophobic) contacts.

## Conclusions and future perspectives

In this study, *Corchorus olitorius* fixed oil was chemically characterized using GC-MS analysis. It was then loaded into polymeric nanoparticles made of a PLGA-PCL combination to create *C. olitorius*/PLGA-PCL NPs. To determine which formulation was the best optimized, 16 formulations were evaluated based on hydrodynamic size, PDI, ϶ potential, and EE%. The best *C. olitorius*/PLGA-PCL NPs showed the lowest size and PDI, maximum surface charge, and greatest EE%. They included 0.6 mL of *C. olitorius* oils, 18 mg of PLGA, and 16 mg of PCL (MCO-F8 formula).

Moreover, they demonstrated a 72-h controlled release of encapsulated *C. olitorius*. With an IC_50_ of 31.25 µg/mL, the *C. olitorius* fixed oil showed strong antiviral activity against the HSV-1 virus, whilst the nanoformulation showed an IC_50_ of 1.95 µg/mL. These results were based on their effects on the antiviral and cytotoxic capabilities. Through protein-protein interactions, a network of 108 genes was created that overlapped HSV-1 targets with genes connected to the *C. olitorius* extract (PPI). Among the genes that showed network connection significance were AKT1, TNF, EGFR, STAT3, SRC, BCL2, IL1B, HSP90AA1, PPARG, and MTOR.

According to the docking study, four fatty acids were found to have strong antiviral activities: 9-hexadecenoic acid, methyl ester, (Z) **(**compound **1)**, *n*-hexadecanoic acid (compound **3**), *cis*−11-eicosenoic acid, methyl ester (compound** 10)**, and methyl 6-*cis*,9-*cis*,11-*trans*-octadecatrienoate (compound** 12**). These results demonstrate the potential of C.O.-loaded NPs as a novel treatment for HSV-1 infections. To confirm the antiviral efficacy of C.O.-loaded NPs in animal models, further investigation is required. To precisely grasp their actions against HSV-1, more research is necessary on the identified critical genes and fatty acids. Enhancing the distribution mechanism of C.O.-loaded NPs may improve their therapeutic effectiveness.

## Data Availability

All data produced or analyzed in this study are included in this article. If any raw data files are required in a different format, they can be obtained from the corresponding author upon reasonable request.

## Abbreviations

EE%: Entrapment efficiency
FT-IR: Fourier transform infrared
GC-MS: Gas chromatography-mass spectrometry
HSV-1: Herpes simplex virus type 1
MNTC: Maximum nontoxic concentration
NPs: Nanoparticles
PCL: Poly(ε-caprolactone) polymers
PDI: Polydispersity index
PLGA: Polylactide-co-glycolide
TEM: Transmission electron microscopy
ZP: Zeta potential
